# Effect of orthodontic premolar extraction on maxillary teeth angulation and arch dimensions in adolescent patients: A 3-D digital model analysis

**DOI:** 10.4317/jced.61064

**Published:** 2024-02-01

**Authors:** Ahmed Abohabib, Maria J. Viñas, Josep M. Ustrell

**Affiliations:** 1 Assistant lecturer of orthodontics KFS university, Egypt. & Ph.D. student, University of Barcelona, Spain; 2 Assistant professor of orthodontics, Complutense University of Madrid, Spain; 3 Full professor of orthodontics, University of Barcelona, Spain

## Abstract

**Background:**

Therapeutic orthodontic premolar extraction is a common orthodontic treatment, yet the extent of its impact on the upper dental arch dimensions and teeth angulations is still under exploration. Hypothesis: We postulated that the therapeutic extraction of orthodontic premolars significantly alters the orientation of teeth and the dimensions of the dental arch. Objective: This study assessed the impact of therapeutic orthodontic premolar extraction on dental arch dimensions and tooth angulations.

**Material and Methods:**

We conducted a retrospective cohort study involving 30 patients who underwent bilateral upper premolar extraction and fixed appliance treatment. Pre and post-treatment dental casts were scanned, and changes in tooth angulations and arch dimensions were evaluated using 3D digital maxillary models. Statistical analyses encompassed the application of paired samples t-test.

**Results:**

Significant post-treatment changes were observed, including distal tipping in anterior teeth, minor mesial tipping in first molars, and a reduction in torque for central incisors and canines. Dental arch dimensions were also influenced, with increased width and depth between the canines and decreased width between the second premolars while inter-molar width and overall arch depth significantly reduced post-treatment.

**Conclusions:**

Therapeutic orthodontic premolar extraction significantly affects dental arch dimensions and tooth angulations. These findings have implications for treatment planning and predicting changes associated with orthodontic treatments involving premolar extraction.

** Key words:**3D digital models - orthodontic premolar extraction - teeth angulation - dental arch dimensions.

## Introduction

Premolar extraction, a common procedure in orthodontics, has been a subject of continuous debate since the early 20th century (1-3). Although it is frequently used to address malocclusions, its potential effects on arch dimensions, tongue position, and palate dimensions have led to discussions (4,5) with research linking dental arch expansion to changes in airway dimensions (6).

To assess orthodontic treatment outcomes, a shift from traditional evaluation methods like cephalometric superimpositions and AutoCAD-based measurements to digital 3D modeling is occurring. Offering several advantages such as durability, ease of sharing, cost reduction, and improved patient education, digital dental models have gained widespread acceptance (7-11). The reliability of measurements obtained from these models has been consistently confirmed by various studies (12,13).

Also, 3D digital models offer comprehensive visual and quantitative analysis of changes post orthodontic treatment, offering a more precise method for assessing dental arch dimension alterations. Created from impressions or direct 3D intraoral scanning, these models eliminate concerns regarding radiation exposure (5,14).

Despite the significant influence of dental arches on facial aesthetics (15) and the potential implications of premolar extraction on arch dimensions, comprehensive evaluations of three-dimensional arch dimensional changes post-extraction using 3D models are scarce.

This study aims to fill these gaps, investigating the effects of therapeutic premolar extraction on upper arch dimensions and tooth positioning, by analyzing three-dimensional models before and after treatment. This research is intended to contribute to a better understanding of the implications of orthodontic premolar extraction, potentially informing future practice and treatment planning.

## Material and Methods

Our retrospective cohort study adhered to The Strengthening the Reporting of Observational studies in Epidemiology (STROBE) guidelines. It aimed to study the effects of extraction treatment on patients through pre- and post-treatment digital dental models.

-Study Design

The study focused on the outcomes of extraction treatment on a group of patients, utilizing digital dental models of pre- and post-treatment.

-Study Setting

We used 30 sets of pre- and post-treatment digital dental models from patients treated at the Orthodontic Clinic of Dental Hospital, University of Barcelona.

-Sample Size

The sample size was determined through calculations based on interfirst molar width parameter of previous study (5) that indicated that the difference in the response of matched pairs is normally distributed with standard deviation 2.8. If the true difference in the mean response of matched pairs is 2.9, we needed to study 10 pairs of subjects to be able to reject the null hypothesis that this response difference is zero with probability (power) 0.8. The Type I error probability associated with this test of this null hypothesis is 0.05. So, the estimated number of participants needed to achieve study objectives was 10 pairs of pre- and post- treatment subjects’ casts, but over sample was done. So, 30 subjects included in the study.

-Participants

Inclusion criteria included late adolescence participants over 16 years old who had completed orthodontic treatment involving upper bilateral first premolar extraction and fixed appliance treatment were used, patients with class II, class I and bimaxillary protrusion malocclusions who were treated with medium or maximum anchorage for closing spaces.

While exclusion criteria included any palatal or dental defects, patients with previous orthodontic treatment, missed anterior permanent teeth, and surgical and dentofacial deformity cases or participants with broken or bad conditions records.

-Exposures

Patients underwent orthodontic treatment involving bilateral therapeutic premolar extraction and fixed orthodontic appliance with 0.022-inch slot size brackets of American Orthodontics was utilized. Additionally, elastics were applied over the 16x22 stainless steel arch wire as sliding mechanics to close the extraction spaces with trans-palatal bar as moderate anchorage appliance.

-Data Sources

Pre- and post-treatment maxillary casts were scanned using the optical 3D dental scanner (3SHAPE E series) lab scanners and virtually constructed. The files were imported into the Nemotec software ((Nemocast. v19.20. Nemotec. Madrid, Spain)).

-Outcome Measurements

The study aimed to assess changes in the upper dental arch, such as arch dimensions, and teeth rotation and inclination so three-dimensional evaluation approach utilizing 3D digital maxillary models were performed. These models underwent a series of steps within the software to enable comprehensive analysis.

**Figure 1:**
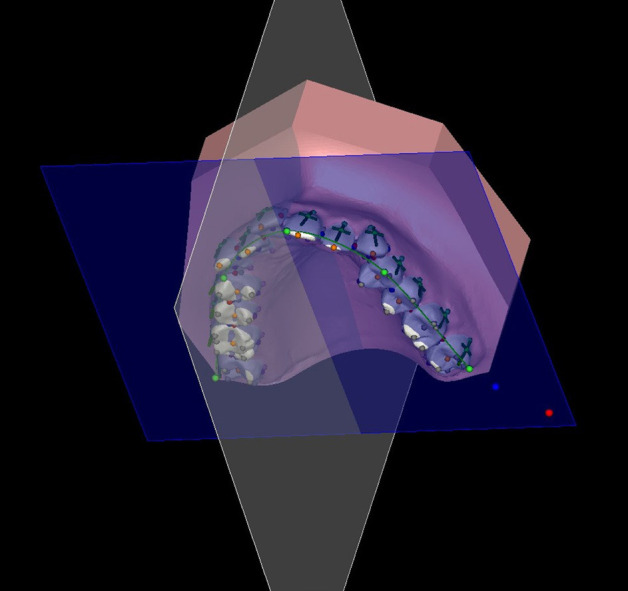
Image displaying teeth landmarks

After integrating the digital models in STL format into the program (A), they were oriented in 3D space through precise repositioning (B). For models lacking digital bases, careful construction was undertaken (C). The teeth were segmented through the identification of tooth centers and radii, as well as the marking of gingival margins and pinpointing of gingival papillae (D). Subsequently, the program automatically placed teeth landmarks, which were further verified by the operator (E). The introduction of an arch form and precise identification of the occlusal plane were performed by the operator (F). With the digital models meticulously set up, we were permitted to extract the essential measurements, (Fig. 1).

**Figure 2:**
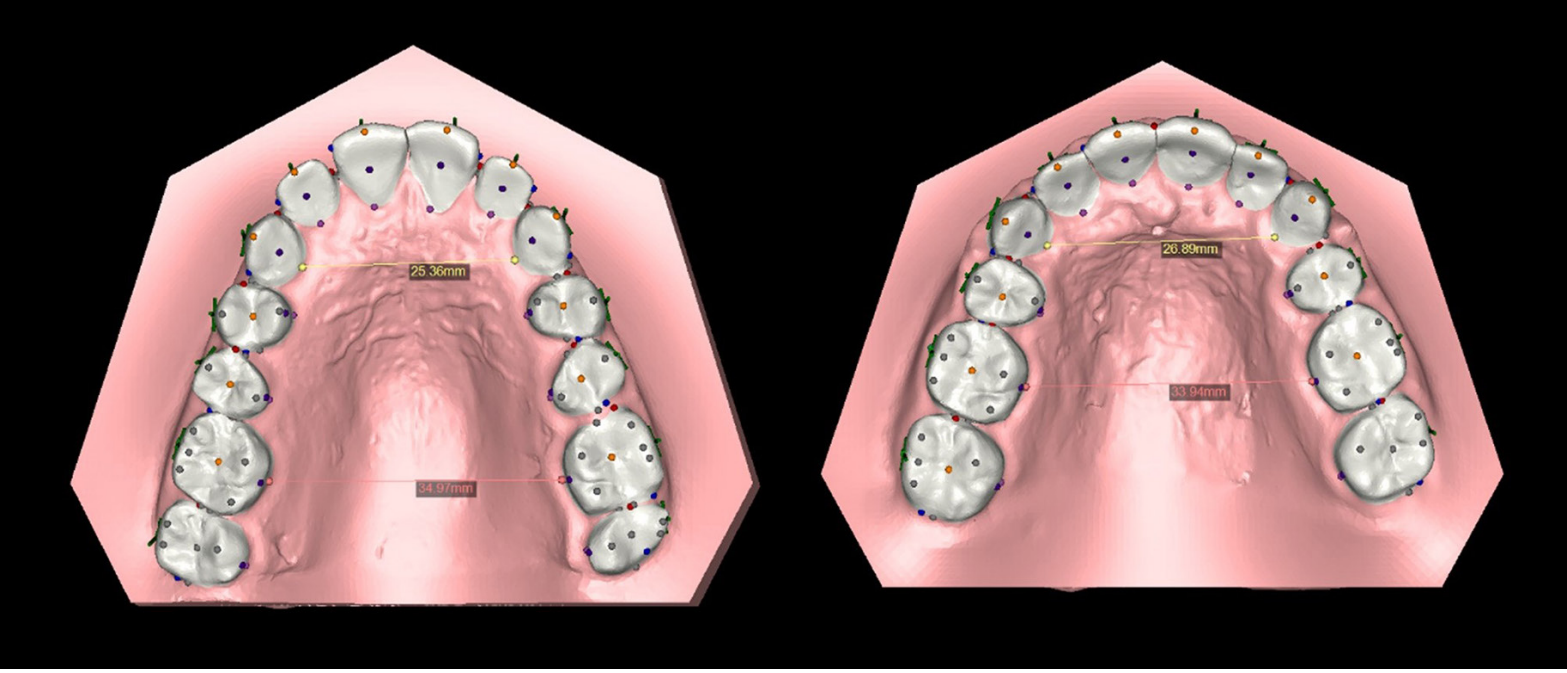
Image displaying some arch dimensions measurements changes, in the left pre-treatment and in the right post-treatment models.

Following segmentation process segmentation process, we extracted pertinent measurements from the 3D digital models both before and after treatment (Fig. 2). The measurements were double checked two times by same examiner to eliminate errors beside to ensure efficient organization and storage, all collected measurements were compiled and exported to an Excel spreadsheet. This facilitated easy comparison and further in-depth analysis.

-Statistical Analysis

Collected data underwent statistical analysis employing SPSS (version 20). Paired samples t-test was utilized. The significance level was established at *P* < 0.05.

-Ethics and Disclosure

Ethical approval was obtained from the Ethics and Research with Medicines and Health Products of Dental Hospital, University of Barcelona (code 19/2019). Data anonymity was maintained.

## Results

Our study focused on the impact of orthodontic premolar extraction on the upper dental arch, assessing changes in teeth angulation and arch dimension. For this purpose, we utilized three-dimensional digital dental models obtained before (T0) and after (T1) the treatment.

After searching through the available database, a list of 78 patients having undergone bilateral extraction of upper premolars who met our study’s inclusion criteria was compiled. However, only 34 patient models could be collected due to certain limitations. Among them, 4 patient models were excluded due to the presence of unerupted anterior teeth in the initial models. Therefore, the final sample for the study consisted of 30 patient models.

**Figure 3:**
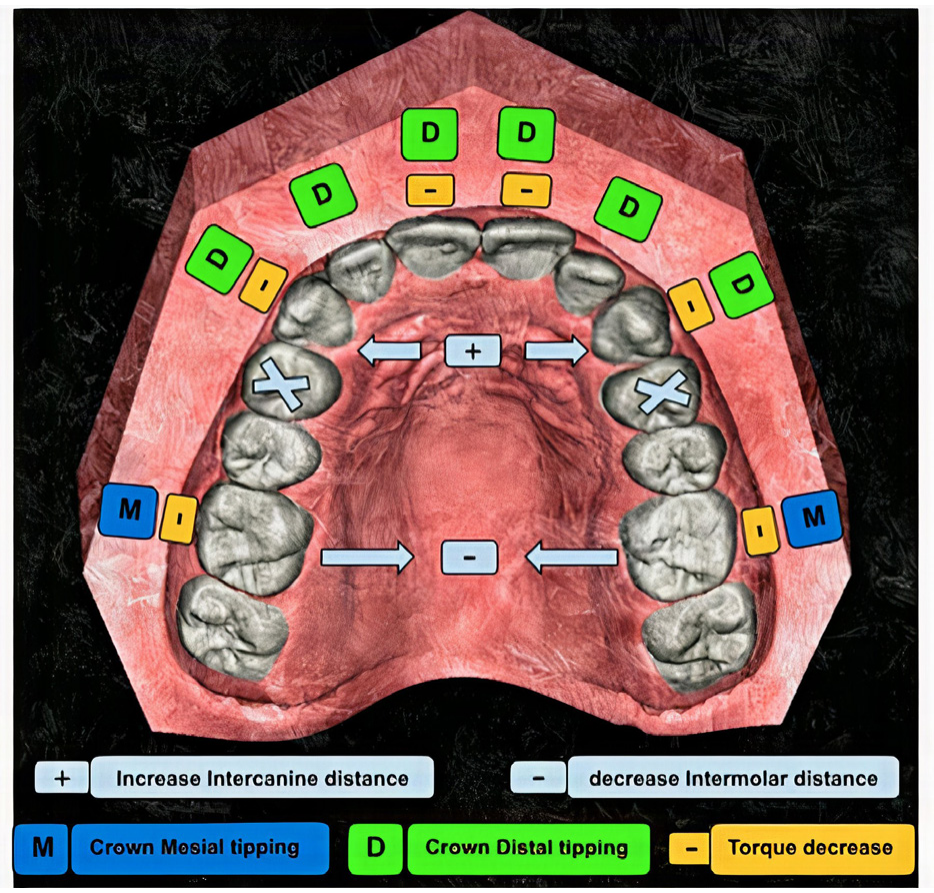
Diagram displaying conclusion of the results.

-Outcome data, (Fig. 3).

In terms of mesiodistal (MD) angulation changes, we found that the anterior teeth, including the maxillary central incisors, lateral incisors, and canines, exhibited a consistent pattern of distal tipping post-treatment. Notably, the lateral incisors and canines demonstrated significant distal tipping changes as follow teeth number 11 and 21 demonstrated significant distal tipping changes (*P* < 0.05) and teeth 12, 22 exhibited more substantial highly significant distal tipping changes (*P* < 0.01). Likewise, teeth 13 and 23 showed a high significant distal tipping change (*P* < 0.01).

In contrast, the maxillary second premolars showed no significant changes in tipping (*P* > 0.05) for teeth number 15 and 25. The first molars exhibited a small degree of mesial tipping for teeth 16 and 26 but this change was not statistically significant (*P* > 0.05). These findings are demonstrated in Table 1.

**Table 1:**
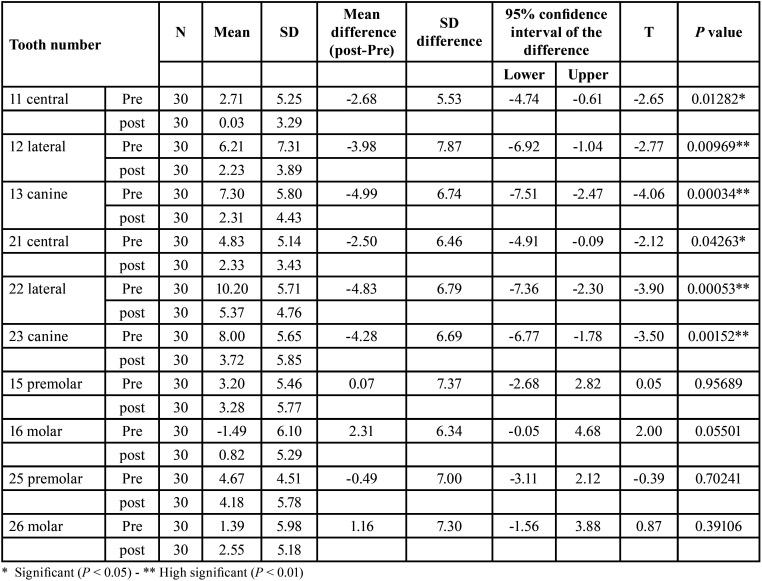
Descriptive statistics & paired samples t-test of teeth MD angle.

In regard to buccolingual (BL) angulation changes, we found that the maxillary central incisors and canines showed significant linguoversion changes, indicating a reduction in torque as teeth number 11 and 21 exhibited significant changes (*P* < 0.05). The maxillary lateral incisors did not show any statistically significant change in torque, implying a sTable position in this respect.

Additionally, the maxillary second premolars did not show any statistically significant change in torque. Conversely, the first molars exhibited a significant decrease in torque. As teeth 16 and 26 showed changes (*P* < 0.05). Detailed information can be found in Table 2.

**Table 2:**
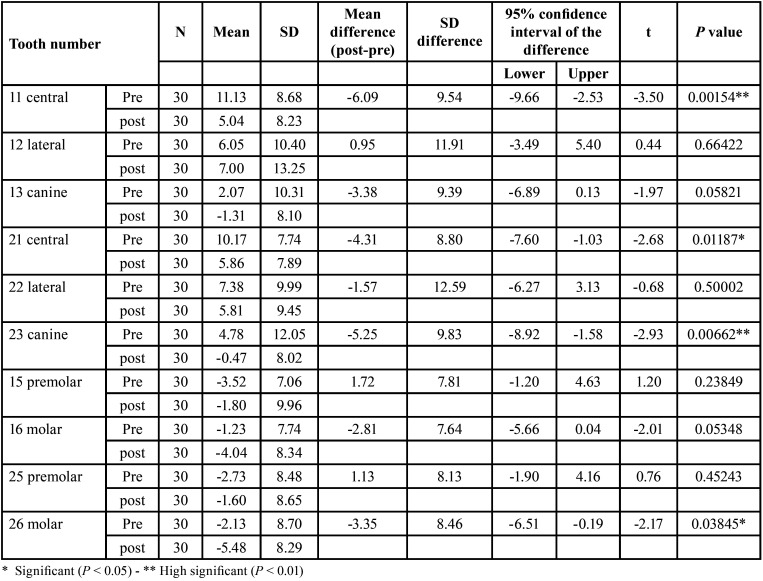
Descriptive statistics & paired samples t-test of teeth BL angle.

While analysis of the arch dimensions, we analyzed various arch variables of width (detailed in Table 3), highlighting the most significant ones we revealed a highly significant increase in both the width and depth between the canines (*P* < 0.01). In contrast, there was a highly significant decrease in the width between the second premolars (*P* < 0.01). These changes indicate that the space between these specific teeth changed noticeably post-treatment.

**Table 3:**
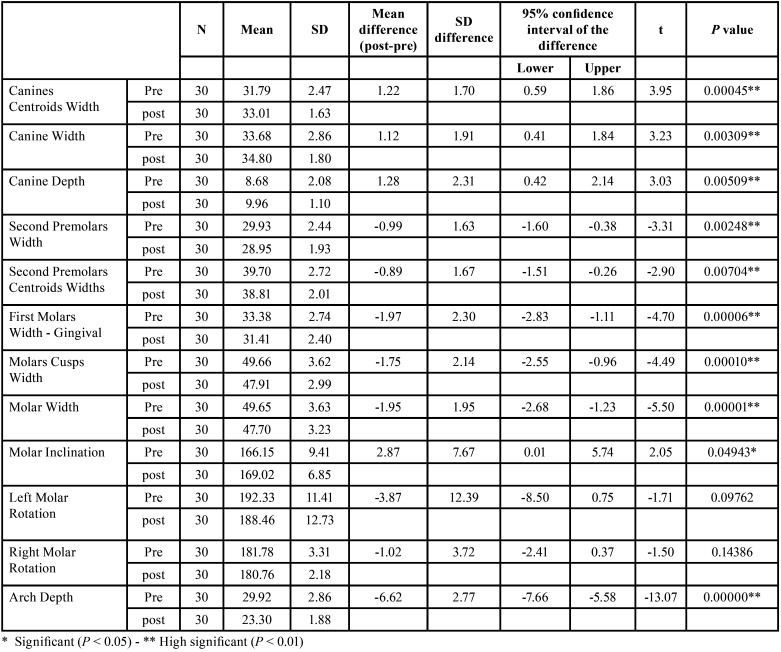
Descriptive statistics & paired samples t-test of arch dimension measurements.

Furthermore, we noted a substantial highly significant reduction in the width between the molars (*P* < 0.001) and the overall arch depth showed a noTable significant reduction in arch depth (*P* < 0.001). However, there was a slight significant change in the inclination of the molars (*P* < 0.05), showing a change in their angle relative to a defined plane, but no significant changes were found in the rotation of the upper right or left molars. These findings are summarized in Table 3.

## Discussion

The realm of orthodontic research continues to embrace technological advancements for improved accuracy and patient safety. One such advancement is the adoption of three-dimensional (3D) digital dental models, which were employed in this study to evaluate the effects of orthodontic premolar extraction on upper arch dimensions, and teeth angulations.

This method demonstrates a substantial upgrade from conventional approaches in the field previous methodologies, such as those used by Narayanan *et al*. (4) that involved collecting photographing of pre- and post-treatment study models. Further measurements of arch dimension alterations were performed using AutoCAD. In contrast, Al Maaitah *et al*. (16) utilized a manual approach, measuring dental cast using a divider and an orthodontic ruler. While historically effective, the 3D digital model which implemented in our study provides a higher degree of accuracy, marking a pivotal step forward in orthodontic research.

Ruan *et al*., (17) on the other hand, used 3D models based on cone-beam computed tomography (CBCT) data for their orthodontic treatment evaluation. Despite its thorough spatial understanding, this method entails a risk of radiation exposure. The potential hazard stresses the necessity for safer data collection methods in orthodontics, like our use of 3D digital models from laser scanning.

The reliability of 3D digital model superimposition in assessing orthodontic tooth movement is well established, as underscored by various research studies (18-21). Also, the study conducted by Cha *et al*. (9) emphasize the importance of three-dimensional digital model superimposition in accurately assessing orthodontic tooth movement. Furthermore, the study by Nambiar *et al*. (22) provides additional support for the use of palatal rugae as dependable landmarks in tracking tooth movement.

Our research findings highlight the significant changes observed in various dental parameters following extraction. We found that therapeutic extraction markedly influences the dynamics of the upper dental arch, particularly in the mesiodistal and buccolingual angulation of anterior teeth, and the arch dimensions. As we noted a consistent distal tipping pattern in the maxillary anterior teeth, in terms of mesiodistal angulation changes. The central incisors, lateral incisors, and canines all demonstrated significant changes in the distal tipping. This observation aligns with Ruan *et al*. (17) report of distal crown tipping of maxillary canines. Such a change can be attributed to the effect of orthodontic retraction forces post premolar extractions, leading to space closure. This result is further supported by Cha *et al*. (23) study, which also noted a tendency for distal tipping of anterior teeth following first premolar extractions.

However, unlike the anterior segment, the maxillary second premolars and first molars did not show significant tipping changes. The first molars exhibited a slight degree of mesial tipping, which was statistically insignificant. This observation aligns with the findings of Kumari and Fida (24) who beside didn’t detect any significant rotation in the maxillary posterior teeth like us. This could suggest that the stability of these teeth may be largely preserved after premolar extraction, potentially due to the firm anchorage provided by orthodontic appliances.

In terms of buccolingual angulation changes, our study demonstrated a significant decrease in torque in the maxillary central incisors and canines, a result in line with findings from previous studies (5,17,25). This change could be an adaptive response to the lingual retraction forces applied during orthodontic treatment, emphasizing the need for careful monitoring and control of torque changes during the treatment process.

Also, our findings indicated absent of changes for maxillary second premolars and minor decrease in torque for first molars and little contracted towards the midsagittal plane, which align with Cho *et al*. results (5), which showed no significant changes in inclination and angulation of second premolars. While first molars showed no significant changes in inclination or angulation, but they differed by reporting displaying mesial rotation of upper molars. The differences underscore the complexity of orthodontic treatments, the need for personalized treatment plans, and the potential impact of particular techniques and appliances.

Our study showed significant alterations in the inter-canine width and depth, as well as between the second premolars and molars post orthodontic treatment. we noticed increase in inter-canine width and depth. Conversely, a significant decrease in the width between the second premolars and the molars. Furthermore, a significant reduction in arch depth and molar width was observed. That was aligning with findings by Meyer *et al*. (26) and Al Maaitah *et al*. (16). Also, same with Kumari and Fida (24) who reported an increase in the maxillary intermolar width in the non-extraction group while the intermolar widths and arch depths decreased in the extraction group and significant increase for the upper inter-canine width but contradicts the findings of Oz *et al*. (27). These changes suggest the dental arch’s dynamic response to biomechanical forces as the increase in inter-canine width and depth could be an outcome of the expansion forces utilized in the treatment to optimize dental alignment and aesthetics, On the other hand, the reduced inter-premolar and inter-molar widths could stem from the closure of space that typically follows extractions.

On the other hand, our results contrast with Nambiar *et al*. study (22), which observed a mean increase in molar area width. Instead, we found a significant decrease in inter-molar width and a reduction in arch depth. Contrary to Gianelly *et al*.’s report (28) that extraction does not result in narrower dental arches, our results, in line with studies by Meyer *et al*. (26) and Narayanan *et al*. (4) founds significant post-extraction decreases in arch width, likely due to the space closure mechanics used.

While this study provides essential insights, certain limitations must be acknowledged. The first pertains to the orthodontic extraction treatment plan used. Specifically, we adopted a moderate anchorage approach during the closure of the extraction space. While Cho *et al*. (5) used second molars banded or bonded in the fixed treatment as moderate anchorage. Some research(17,29) utilized maximum anchorage methods, like mini screws, while others like Haque *et al*. (30) employed no anchorage during the retraction of anterior teeth and closure of extraction space. However, different anchorage techniques can lead to varied tooth movement, influencing the overall treatment outcome and, potentially, alterations in arch dimensions.

The study also exclusively focused on patients who had premolar extractions, there by lacking a non-extraction comparison group.

The findings of this study significantly contribute to understanding the impact of premolar extraction on upper dental arch dynamics. The results obtained underscore the need for future research to include both extraction and non-extraction groups and to consider the effects of varying anchorage methods. This comprehensive approach can provide a deeper understanding of the interplay between these variables and their subsequent impact on the morphology of the palatal vault, ultimately improving patient outcomes in orthodontic treatment by better understanding the expected changes in arch dimensions and teeth angulations following extraction treatment.

## Conclusions

• Extraction of orthodontic premolars had a significant impact on dental arch dimensions and tooth positioning, causing marked changes in angulation of anterior teeth and inter-canines, premolars, and molars’ arch dimensions.

• Consistent distal tipping in the maxillary anterior teeth were observed. Meanwhile, maxillary second premolars remained relatively stable throughout the treatment process.

• In terms of buccolingual angulation changes, central incisors and canines exhibited linguoversion, signifying a substantial decrease in torque following treatment. Additionally, a slight increase in lingual crown inclinations of molars was observed.

• Alterations in arch dimensions manifested as an increase in inter-canine width, while a noTable reduction in inter-molar width and overall arch depth. This reduction indicates a noteworthy narrowing and reduction in arch depth post-extraction.
